# Compulsive Exercise and Changes in Physical Activity Among Females: A Longitudinal Analysis During the First 12 Months of the COVID‐19 Pandemic

**DOI:** 10.1002/brb3.70456

**Published:** 2025-04-01

**Authors:** Hannah J. White, Chris J. McLeod, Emma Haycraft, Carolyn R. Plateau, Clare E. Holley, Gemma L. Witcomb

**Affiliations:** ^1^ School of Sport, Exercise and Health Sciences Loughborough University Loughborough UK

**Keywords:** body image, coronavirus, transitions, weight concerns

## Abstract

This study had two aims; first to explore changes in compulsive exercise among adult females across the first year of the COVID‐19 pandemic, and second, to explore how changes to physical activity early in the pandemic impacted compulsive exercise.

Participants were 174 adult females who completed online surveys four times (T1–T4) during the first 12 months of the pandemic. Participants reported changes in physical activity (T1) and completed the compulsive exercise test (CET) at each time point (T1–T4).

Mixed ANOVAs with time (T1–T4) and group (did versus did not report changes in physical activity) as factors revealed that compulsive exercise significantly differed across timepoints for the whole sample. Furthermore, females who reported that their physical activity levels had changed early in the pandemic reported higher levels of CET Weight Control Exercise compared to those who reported no change. No significant interactions were found.

Among this sample of adult females, attitudes towards exercise changed during the first year of the pandemic, which may reflect the impact of varying lockdown restrictions. Additionally, changes to physical activity early in the pandemic were linked with exercising to control body weight. This suggests that externally influenced changes in physical activity may be an influential factor in the development of compulsive exercise. This may have implications for understanding and managing risk in a range of transition situations that impact upon physical activity.

## Graphical abstract

One hundred and seventy‐four females reported on whether their physical activity changed at the start of the pandemic, and their exercise behaviors at four time points across 12 months. Results from the Compulsive Exercise Test showed that exercise for weight control was significantly higher in females who reported a change in physical activity early in the pandemic (n = 104) compared to those who did not (n = 70).

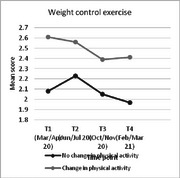

## 1 Introduction

In response to the COVID‐19 pandemic, on March 23, 2020, the British government issued a “stay at home” order (Gov.uk 2020). This led to the closure of all non‐essential businesses, including schools, gyms, and sports facilities. Alongside this, multiple statements and articles were released about the importance of staying active during the pandemic (e.g., Chen et al. 2020; Dwyer et al. 2020; Stamatakis et al. 2020; WHO 2020). Recommendations about exercising at home were also released with examples of how to adapt activities to continue to be active (e.g., climbing stairs or doing an online exercise class; American College of Sports Medicine 2020; Gov.uk 2020; WHO 2020; for a review of recommendations see Polero et al., 2021).

Despite this information, early cross‐sectional research found that physical activity levels were impacted by the pandemic, with most research reporting an initial reduction in activity levels (e.g., Strain et al. 2022; for reviews see Caputo and Reichert 2020; Stockwell et al. 2021). However, some studies reported the opposite, with some adults reporting exercising at the same level or more compared to before the pandemic (Phillipou et al. 2020; Robinson et al. 2021). Thus, this suggests that the pandemic had a variable impact on individuals, and affected physical activity levels differently. While a number of factors may influence changes in physical activity (e.g., pre‐pandemic activity levels; Brand et al. 2020), two important factors are being female (Nienhuis and Lesser 2020; Robertson et al. 2021) and having a current or previous mental health condition; specifically, having a current or past diagnosis of an eating disorder (ED) (e.g., Robertson et al. 2021; Stockwell et al. 2021). The links between exercise and eating psychopathology are well acknowledged (e.g., Meyer et al. 2011).

Concerns were highlighted early in the pandemic regarding how disruptions to physical activity routines may impact ED risk via increased shape and weight concerns or adopting alternative unhealthy compensatory behaviors (Cooper et al. 2020; Rodgers et al. 2020). Related to these concerns was the notable increased focus on body weight during the pandemic, particularly within the media. For example, articles/posts discussed increased food intake and reduced exercise leading to weight gain—dubbed the ‘Quarantine 15’ (Pearl 2020). Alongside this were increased perceptions of the importance of being active during this time (Sport England 2020), emphasized by regular discussions about exercise within daily UK COVID‐19 government updates and increased activity‐based content available online (Branley‐Bell and Talbot 2020; Vuillier et al. 2021). This suggests a strong societal narrative relating to being active and body weight and shape, potential fueling concerns. This may have been further intensified by perceived positive changes to others’ physical activity levels, particularly as the pandemic was often framed as a time for “transformation” in relation to body weight and fitness (Vuillier et al. 2021 9). For example, Breiner et al. (2021 5) found that more participants reported “pressure to get in shape” as a motive for exercise during the pandemic compared to before.

An important component of eating psychopathology aetiology, development, and maintenance is compulsive exercise (CE; Meyer et al. 2011). This is a multidimensional concept based on a “highly driven urge to be active” (Meyer et al. 2016 1). It transcends beyond exercise as a method of weight control alone to also include emotional and cognitive aspects, such as rigidity around exercise, lack of enjoyment, avoidance of negative withdrawal symptoms, and positive reinforcement through mood improvement (Taranis et al. 2011). In line with this, physical activity/exercise limitations during the pandemic may have presented specific risk factors for disordered eating, such as potential withdrawal symptoms, restrictive eating, increased psychological distress (Cooper et al. 2020), and heightened shape and weight concerns (Rodgers et al. 2020). For example, individuals with an ED who reported an exacerbation of their symptoms during the pandemic reported changes to physical activity as a key factor (Vuillier et al. 2021), highlighting the interconnected nature of exercise and eating psychopathology.

While several studies have reported a worsening of ED symptoms during the pandemic (see Linardon et al. 2022 for a review), a meta‐analysis by Sideli et al. (2021) found no significant differences in symptoms between pre‐pandemic and the first lockdown based on longitudinal studies. However, less is known about CE specifically. Research with clinical samples has highlighted specific challenges with exercise during the pandemic. For example, many individuals with experience of an ED reported exercising more (e.g., Phillipou et al. 2020) and thinking about exercise more (Robertson et al. 2021; Sideli et al. 2021). Such individuals also reported difficulties in managing “their relationship with exercise during lockdown” (Branley‐Bell and Talbot 2020 5) and anxieties about not being able to exercise (Termorshuizen et al. 2020), with many reporting “weight” as a motive to exercise (Breiner et al. 2021). While these findings broadly align with different aspects of CE, there is limited research specifically exploring CE during the pandemic within a community sample.

Initial research using a single item measure of CE suggested no significant change in CE or ‘compensatory physical exercise’ compared to pre‐pandemic among healthy controls (i.e., no history of EDs) or a community sample (based on retrospective reports; Breiner et al. 2021; Castellini et al. 2020), but an increase in ‘driven exercise’ among college females (Zhou and Wade 2021; although samples were not matched). However, these studies focused on the frequency of driven exercise episodes in relation to controlling body weight and shape, not the broader cognitive‐behavioral aspects of CE that have been previously identified (e.g., mood regulation; Taranis et al. 2011). Recent findings reported a different pattern, with higher levels of both CE and weight and shape exercise during the pandemic compared to before, based on a case‐control matched sample study (Palermo and Rancourt 2022). Together, the limited research exploring CE with community samples during the pandemic presents mixed findings. Furthermore, data from a cross‐cultural study found that participants at risk of the highest levels of problematic or excessive exercise were from the UK (Dores et al. 2021). However, no research has explored CE in a general sample population from the UK during *different* points of the pandemic, when access to amenities and activities changed due to various government‐imposed restrictive orders. Additionally, Schneider et al. (2023) discuss how a change in physical activity may have impacted ED behaviors, such as excessive or compulsive exercise, yet little is known about this in relation to CE during the pandemic.

In summary, many adults report that the COVID‐19 pandemic has led to changes in physical activity levels (e.g., Stockwell et al. 2021). However, although early concerns were raised (Rodgers et al. 2020), little is known about how CE may have varied during the first year of the pandemic and whether any changes to physical activity levels may have impacted CE during the pandemic. It has also been outlined that females may have experienced particular challenges in relation to both eating psychopathology and exercise during the pandemic (e.g., Mikhail 2023; Robertson et al. 2021; Taquet et al. 2022). Therefore, the current study aims to explore any changes in CE over the first year of the pandemic and investigate the impact of changes in physical activity on CE among adult females.

## 2 Methods

### 2.1 Participants

One hundred and seventy‐four females aged 18 to 65 (mean age = 36.6 years, SD = 14.12) participated. BMI scores were calculated based on self‐reported height and weight (N = 166; mean BMI = 24.9, SD = 4.72). Participants were predominantly White British/other (92.4%), cisgender (99.4%), with the majority employed full time (47.7%) or part time (16.7%) or studying full time (23%) before the COVID‐19 pandemic began. A number of participants reported currently or previously receiving mental health treatment (30.6%) or treatment for an eating disorder (6.4%).

### 2.2 Recruitment/Procedure

Participants were recruited through a university press release and social media via opportunity sampling. After ethical approval was obtained (via Loughborough University Ethics Review Sub‐Committee; 2020–1378–181), and consent was provided, participants were invited to complete an online survey at three time points during the COVID‐19 pandemic (T1: 28 March– April 30, 2020, T2: 02 June– July 01, 2020, T3: 15 October– November 15, 2020). As the pandemic continued, a fourth time point (T4: 15 February– March 16, 2021) was added to extend the longitudinal analysis to 12 months. Participants were invited to take part at each time point having completed T1 (see supplementary materials (Table S1) for a summary of government restrictions during the four time points and how these relate to physical activity facilities). Two voucher prize draws took place for participants who completed all three initial time points, and everyone who completed T4. Only participants who completed all four of the study time points were included in the analyses (N at T1 = 580; 30% completed all time points). This sample and dataset are part of a larger study exploring health and wellbeing across the first year of the pandemic (Witcomb et al. 2023); the current study focused on the exercise data.

### 2.3 Measures

Each online survey included demographic questions and the measures below.

#### 2.3.1 Leisure Time Physical Activity

At all timepoints, participants completed the validated Leisure Time Exercise Questionnaire (LTEQ; Godin and Shephard 1997). Participants were asked to indicate how many times on average they had done a) strenuous, b) moderate, and c) mild (minimum effort) exercise for more than 15 minutes during their free time over the past seven days (one week). Total weekly leisure activity was calculated by multiplying weekly frequencies of strenuous, moderate, and mild activities by nine, five, and three, respectively, before summing the products of the separate components. Higher scores indicate greater levels of physical activity.

#### 2.3.2 Changes to Physical Activity

At time point 1, after completing the LTEQ, participants were asked, “Is the last 7 days typical of your normal physical activity BEFORE the COVID‐19/Coronavirus pandemic (e.g., before 1st March 2020)?” Response options were yes or no. Questions relating to changes in exercise behaviors within the previous week compared to before the COVID‐19 pandemic have been used in other published studies (e.g., Phillipou et al., 2020).

#### 2.3.3 Compulsive Exercise

At all time points, participants completed the 24‐item Compulsive Exercise Test (CET) (Taranis et al. 2011). This measure is comprised of five subscales: Avoidance and Rule‐Driven Behavior (eight items), Weight Control Exercise (five items), Mood Improvement (five items), Lack of Exercise Enjoyment (three items), and Exercise Rigidity (three items). The CET uses a six‐point Likert scale anchored by 0 (never true) and 5 (always true), with higher scores indicative of greater pathology. Subscale scores were based on the mean score of items. Reliability in the current sample was acceptable for Exercise Rigidity (α 0.74‐0.77 across time points) and very good for all other subscales (α ≥ 0.83).

### 2.4 Data Analysis

Data analysis was conducted in SPSS version 27 (SPSS Inc., Somers, NY). A series of mixed ANOVAs with time (T1, T2, T3, T4) and group (no change, changes in physical activity) as factors were conducted to test the study aims. Where sphericity was violated (Mauchly's test p< 0.05) and there was an increased probability of a Type‐2 error, Huynh‐Feldt (1976) estimates were included. Post‐hoc pairwise comparisons were conducted on any significant main effects, using Bonferroni's correction. Due to the exploratory nature of this study the alpha level was set at p< 0.05. Tests of difference between those who completed all four time points versus those who dropped out highlighted significant differences in age (completers mean = 36.6 years; non‐completers mean = 33.1 years; Z = 2.93, *p* = 0.003), but no significant differences for BMI (*p* = 0.297), or compulsive exercise scores (*p* ≥ 0.212) at T1.

## 3 Results

### 3.1 Descriptive Statistics

One hundred and four participants (59.8%; mean age = 34.3 years (SD = 13.1); mean BMI = 25.3 (SD = 4.8)) reported that their physical activity changed early in the pandemic, whereas 70 participants (40.2%; mean age = 40.1 years (SD = 14.9); mean BMI = 24.4 (SD = 4.6) reported that their physical activity had not changed.

Mean scores for the CET for (1) the whole sample, (2) those who reported a change in their physical activity early on in the pandemic, and (3) those who did not report a change, are shown in Table 1.

**TABLE 1:** Descriptive statistics (means and SDs) for compulsive exercise subscales for the whole sample (N = 174), females who reported a change in physical activity early on in the pandemic (n = 104), and those who reported no change in physical activity (n = 70).

	Whole sample N = 174				Change in physical activity N = 104				No change in physical activity N = 70			
	**T1** **Mean (SD)**	**T2** **Mean (SD)**	**T3** **Mean (SD)**	**T4** **Mean (SD)**	**T1** **Mean (SD)**	**T2** **Mean (SD)**	**T3** **Mean (SD)**	**T4** **Mean (SD)**	**T1** **Mean (SD)**	**T2** **Mean (SD)**	**T3** **Mean (SD)**	**T4** **Mean (SD)**
**CET Scores**												
Avoidance and rule‐driven behavior	1.72 (1.15)	1.66 (1.14)	1.65 (1.18)	1.62 (1.12)	1.81 (1.11)	1.79 (1.17)	1.79 (1.22)	1.77 (1.19)	1.59 (1.22)	1.47 (1.07)	1.44 (1.10)	1.41 (0.99)
Weight control exercise	2.40 (1.16)	2.43 (1.16)	2.25 (1.18)	2.23 (1.16)	2.61 (1.14)	2.56 (1.15)	2.39 (1.21)	2.41 (1.24)	2.08 (1.13)	2.23 (1.16)	2.05 (1.10)	1.97 (0.98)
Mood Improvement	3.29 (1.14)	3.05 (1.14)	3.04 (1.20)	3.08 (1.16)	3.36 (1.02)	3.14 (1.13)	3.15 (1.18)	3.20 (1.08)	3.18 (1.30)	2.93 (1.15)	2.87 (1.23)	2.90 (1.26)
Lack of exercise enjoyment	1.78 (1.25)	1.90 (1.29)	1.87 (1.32)	1.99 (1.31)	1.71 (1.09)	1.83 (1.17)	1.79 (1.23)	1.98 (1.16)	1.88 (1.46)	2.01 (1.45)	2.00 (1.45)	2.01 (1.51)
Exercise Rigidity	2.76 (1.26)	2.61 (1.19)	2.42 (1.23)	2.50 (1.20)	2.91 (1.10)	2.65 (1.16)	2.43 (1.19)	2.57 (1.10)	2.53 (1.44)	2.54 (1.25)	2.41 (1.30)	2.39 (1.33)

Note: CET = Compulsive Exercise Test (Taranis et al. 2011).

### 3.2 Physical Activity Levels

A preliminary ANOVA outlined that there was a significant main effect of time on physical activity levels (*F* [2.90, 490.69] = 4.23, *p* = 0.006, n^2^_p_ = 0.024). Post‐hoc pairwise comparisons highlighted that this was the result of participants reporting lower levels of physical activity at T3 (44.64) compared to T2 (52.02; *p* = 0.013). There was no significant main effect for group (*F* [1, 169] = 1.70, *p* = 0.20) or interaction of time and group (*F* [2.90, 490.69] = 0.61, *p* = 0.60).

### 3.3 Compulsive Exercise

As shown in Table 2 (and the online supplementary materials (Figure S1)), the results of the mixed ANOVAs show a significant main effect of time for Weight Control Exercise for the whole sample. Post‐hoc comparisons highlighted that this was the result of participants reporting significantly lower levels of exercise for weight control reasons at T3 (2.22; *p* = 0.013) and T4 (2.18; *p* = 0.003) compared to T2 (2.39). Similarly, a significant main effect of time was shown for Mood Improvement, with participants reporting significantly lower levels of exercise for mood improvement at T2 (3.02; *p = 0*.001), T3 (2.99; *p < 0*.001) and T4 (3.03; *p* = 0.007), compared to T1 (3.27). A significant main effect of time was also shown for Lack of Exercise Enjoyment. Participants reported significantly greater lack of exercise enjoyment at T2 (1.94; *p* = 0.036) and T4 (2.00; *p* = 0.006) compared to T1 (1.79). A final significant main effect of time was shown for Exercise Rigidity, with participants reporting significantly lower levels of exercise rigidity at T3 (2.42; *p* = 0.002) and T4 (2.48; *p* = 0.033) compared to T1 (2.72). No significant main effect of time was found for Avoidance and Rule‐Driven Behavior.

**TABLE 2:** Results of mixed ANOVAs exploring the effects of time (T1, T2, T3, and T4) and group (changes in physical activity (n = 104) versus no changes in physical activity (n = 70)) on compulsive exercise among adult females (N = 174).

	4×2 ANOVA (Main effect of time)		4×2 ANOVA (Main effect of group)		4×2 ANOVA (Interaction)	
	F value	P value	F value	P value	F value	P value
**CET Scores**						
Avoidance and rule‐driven Behavior	*F* [3, 501] = 2.32	0.075	*F* [1, 167] = 3.52	0.062	*F* [3, 501] = 0.89	.446
Weight control exercise	*F* [3, 504] = 6.15	**< 0.001**	*F* [1,168] = 6.08	**0.015**	*F* [3, 504] = 1.23	.299
Mood improvement	*F* [3, 498] = 6.99	**< 0.001**	*F* [1, 166] = 2.63	0.107	*F* [3, 498 = 0.37	.777
Lack of exercise enjoyment	*F* [2.86, 472.47] = 4.43	**0.005**	*F* [1, 165] = 0.96	0.328	*F* [2.86, 472.47] = 0.69	.552
Exercise rigidity	*F* [2.86, 489.00] = 5.90	**< 0.001**	*F* [1, 171] = 1.07	0.303	*F* [2.86, 489.00] = 2.15	.096

Note: Bold text indicates significant main effect.

There was a significant main effect of perceived early changes to physical activity in relation to Weight Control Exercise. Post‐hoc pairwise comparisons highlighted that higher levels of exercising for weight control reasons were reported by participants who reported changes to their physical activity early on in the pandemic compared to those who did not (2.49 versus 2.08; *p* = 0.015). No significant main effects of group were reported for Avoidance and Rule‐Driven Behavior, Mood Improvement, Lack of Exercise Enjoyment or Exercise Rigidity.

There were no significant interactions between time and group for any of the CET subscales.

## 4 Discussion

The first aim of the current study was to explore changes in compulsive exercise among adult females over the first year of the COVID‐19 pandemic. The second aim was to explore how changes in physical activity were related to compulsive exercise during this time. Our results showed changes in all subscales of compulsive exercise for the whole sample across the 12 month period, with the exception of Avoidance and Rule‐Driven Behaviors. Generally, this reflected higher levels of exercising to improve mood and higher levels of rigidity related to exercise at the start of lockdown which then decreased over time. Exercising to control weight differed slightly and was at the highest point at the second time point (June‐July 2020; a period of reduced restrictions), compared to later in the 12 month period of the study. This likely reflects the increased opportunities to exercise at that time, but could also suggest that motives to exercise may have been appearance‐related perhaps due to concerns about increasing in‐person social interactions as restrictions relaxed. Compulsive exercise has been shown to mediate the relationship between fear of negative appearance evaluations and wellbeing among Polish participants during the pandemic, supporting the connection between appearance concerns and CE at this time (Novita et al. 2022). Overall, the current findings suggest that attitudes and behaviors related to exercise likely changed early in the pandemic, with lockdown increasing the focus on exercising for mood regulation and enhanced rigidity around exercise, but that over time this focus reverted to being less pathological, with no lasting increases in compulsive exercise. It should be noted, however, that feeling less enjoyment from exercise increased over the 12‐month period, remaining elevated at the end of the study. This may reflect feelings towards the varying restrictions (i.e., regional (T3) and national lockdowns (T3 and T4)) and the continued reduced access to sports facilities and activities.

This is the first study to explore compulsive exercise levels multiple times within a 12 month period within a community sample. While seasonal fluctuations in physical activity typically occur (Strain et al. 2022; also found within the current study), less is known about compulsive exercise. Due to the nature of compulsive exercise (i.e., a drive to exercise), consistency across seasons was expected. However, this was not found within the current study, and it is likely that the changes reported here have been exacerbated by the fact that the summer months coincided with restrictions easing (T2; June–July 2020) and the autumn (T3; October–November 2020) coincided with regional and then national lockdowns. Evidence is starting to emerge of seasonality in relation to body dissatisfaction and dieting behaviors (e.g., Griffiths et al. 2021; Griffiths et al. 2022); it is possible that the data presented here reflects the exercise behaviors and attitudes that accompany this. Due to the established links between eating psychopathology and compulsive exercise (Meyer et al. 2011), further research should explore the ‘seasonality’ of compulsive exercise in relation to dieting and body dissatisfaction across a 12‐month period outside of pandemic restrictions.

When accounting for whether participants reported changes to physical activity early in the pandemic or not, significant differences were found for compulsive exercise in relation to weight control exercise, with higher levels among those who reported an early change, compared to those who did not. Rodgers et al. (2020) raised initial concerns regarding how disruptions to physical activity/exercise routines during the pandemic may be a risk factor for disordered eating, and specifically shape and weight concerns. Schneider et al.'s (2023) systematic review expands on this to suggest that a change in physical activity may impact ED behaviors related to exercise. The findings of the current study provide support for these initial concerns and additionally suggest such changes may also lead to increased exercising for weight control. This again reinforces the interrelated nature of eating and exercise psychopathology. The importance of discussing pandemic‐related changes to physical activity routines with patients has been raised as a recommendation for eating disorder clinicians (Schneider et al. 2023) and the current findings suggest that this may be an important topic for health professionals working with the general population too. This is of particular importance due to the recognized relationships between CE and quality of life (Meneguzzo et al. 2022).

Despite a single question being used previously to assess changes to physical activity/exercise behaviors during the pandemic (e.g., Phillipou et al. 2020), there are limitations to this approach (e.g., Diamantopoulos et al. 2012). Additionally, within the current study, the response options did not ascertain the way in which participants’ physical activity had changed early in the pandemic (i.e., increased or decreased, or whether there was a change in the type or intensity of activities being completed), merely whether their physical activity was at that time typical for them or not. It is important to note that groups did not significantly differ in their reported PA levels. In line with governmental restrictions closing gyms and sports facilities and recommendations to remain active (e.g., WHO 2020), it could be expected that physical activity and exercise routines were adapted in different ways. This suggests that ‘change’ may not be as simple as an increase or decrease in physical activity alone as often reported in previous research (e.g., Phillipou et al. 2020). Subsequently, the findings suggest that *any* perceived change to physical activity routines may be related to increased exercise attitudes related to weight control. The findings of this research suggest further exploration is needed regarding the ways in which activity behaviors may have changed over the pandemic and how these different changes may relate to compulsive exercise.

Strengths of this novel longitudinal research include that it comprises four data time points exploring exercise pathology across the first 12 months of the pandemic. The community sample includes females of a wide age range and both those with and without experience of an eating disorder. However, it is important to acknowledge that the sample does lack diversity, particularly in terms of ethnicity, which when coupled with the widely acknowledged challenges of participant retention for longitudinal research (Gustavson et al. 2012), could limit the generalizability of findings and overlooks the impact of important intersectional influences (Mandelbaum 2020). Additionally, the lack of baseline pre‐pandemic data is also a limitation. Without this data, it cannot be known whether the onset of the pandemic may have elevated compulsive exercise scores which have then remained increased.

In summary, the current findings of this longitudinal study suggest that among adult females, attitudes and behaviors related to exercise changed during the first year of the pandemic, which likely reflects the enforced varying restrictions. Additionally, changes to physical activity early in the pandemic appear to be linked with greater exercise for weight control. This suggests that some females may need support on how to manage their relationship with exercise during transitional periods, in relation to their body‐related concerns. Resources to support such transitions are needed and should be developed with ease of accessibility in mind (i.e., digital access). While this study is grounded within the context of pandemic‐enforced changes, it is important to note that other transitional periods may have similar effects (for example, transitions within living arrangements, job roles, identities, seasonality). Thus, this study adds critical insight into how transitions more generally may affect compulsive exercise behaviors and reinforces that longer‐term support may be needed regarding how to manage any future transitions in activity. The study highlights the need for support resources related to managing transitions within exercise.

## Author Contributions

**Hannah J. White**: conceptualization, investigation, writing–original draft, methodology, formal analysis. **Chris J. McLeod**: conceptualization, investigation, methodology, writing–review and editing, data curation. **Emma Haycraft**: conceptualization, methodology; writing–review and editing. **Carolyn R. Plateau**: conceptualization, investigation, methodology, writing–review and editing. **Clare E. Holley**: conceptualization, investigation, writing–review and editing, methodology. **Gemma L. Witcomb**: conceptualization, investigation, methodology, writing–review and editing, supervision, data curation.

### Peer Review

The peer review history for this article is available at https://publons.com/publon/10.1002/brb3.70456

## Supporting information



Supporting Information

## Data Availability

Research data are not shared.
