# Supplementary material for: Compulsive Exercise and Changes in Physical Activity Among Females: A Longitudinal Analysis During the First 12 Months of the COVID‐19 Pandemic
Source: Brain Behav. 2025 Apr 1;15(4):e70456. doi: 10.1002/brb3.70456 (PMC11962212; doi:10.1002/brb3.70456)
Supplement: Supplementary file 1 — Supporting Information [file BRB3-15-e70456-s001.docx]

**Supplementary Materials: Compulsive exercise and changes in physical activity among females: A longitudinal analysis during the first 12 months of the COVID-19 pandemic.**

Table S1: Timepoint dates and a summary of Government restrictions in England at those times, including how these related to physical activity facilities (Institute for Government, 2022; Sport England, 2021; UK active, n.d.).

| Timepoint dates | Summary of Government restrictions in England |
| --- | --- |
| Timepoint 1:  28 March – 30 April 2020 | Government enforced lockdown. Stay at home order. Sports facilities and gyms closed. Outdoor exercise permitted once a day. |
| Timepoint 2:  2 June – 1 July 2020 | Lockdown restrictions eased (including phased reopening of schools (1 June); relaxing of restrictions and social distancing rule (23 June). Indoor gyms remain closed. |
| Timepoint 3:  15 October – 15 November 2020 | Regional tiered Government restrictions, with a second national lockdown enforced on 5 November. Gyms open until lockdown with restrictions for indoor team sports. After lockdown, all indoor and any organised outdoor activities are restricted and gyms closed. |
| Timepoint 4:  15 February – 16 March 2021 | Third national lockdown began on 6 January, indoor and outdoor sport facilities closed. Stay at home order remains in place (apart from for essential activities) but restrictions begin to ease with schools reopening on 8 March and recreation permitted between two people in an outdoor public place. |

Figure S1: Graphs highlighting changes in compulsive exercise (CET) scores for T1 to T4 among adult females who reported a change in physical activity early in the COVID-19 pandemic (n=104) and females who did not (n=70).
